# Bacteriophages: Underestimated vehicles of antibiotic resistance genes in the soil

**DOI:** 10.3389/fmicb.2022.936267

**Published:** 2022-08-04

**Authors:** Yue Zhang, Yajie Guo, Tianlei Qiu, Min Gao, Xuming Wang

**Affiliations:** Beijing Key Laboratory of Agricultural Genetic Resources and Biotechnology, Institute of Biotechnology, Beijing Academy of Agriculture and Forestry Sciences, Beijing, China

**Keywords:** bacteriophages, antibiotic resistance genes, mobile antibiotic resistome, horizontal gene transfer, soil

## Abstract

Bacteriophages (phages), the most abundant biological entities on Earth, have a significant effect on the composition and dynamics of microbial communities, biogeochemical cycles of global ecosystems, and bacterial evolution. A variety of antibiotic resistance genes (ARGs) have been identified in phage genomes in different soil samples. Phages can mediate the transfer of ARGs between bacteria *via* transduction. Recent studies have suggested that anthropogenic activities promote phage-mediated horizontal gene transfer events. Therefore, the role of phages in the dissemination of ARGs, which are a potential threat to human health, may be underestimated. However, the contribution of phages to the transfer of ARGs is still poorly understood. Considering the growing and wide concerns of antibiotic resistance, phages should be considered a research focus in the mobile resistome. This review aimed to provide an overview of phages as vehicles of ARGs in soil. Here, we summarized the current knowledge on the diversity and abundance of ARGs in soilborne phages and analyzed the contribution of phages to the horizontal transfer of ARGs. Finally, research deficiencies and future perspectives were discussed. This study provides a reference for preventing and controlling ARG pollution in agricultural systems.

## Introduction

With the extensive use of antibiotics in clinics and agriculture, the problem of antibiotic resistance has become increasingly prominent and has become a major public health risk faced by human society in the 21st century (Kavagutti et al., 2019; Pu et al., 2020). According to the latest report by the *Lancet*, there were an estimated 4.95 million deaths associated with bacterial antimicrobial resistance in 2019, including 1.27 million deaths attributable to bacterial antimicrobial resistance (Murray et al., 2022). If it is not effectively controlled, it is expected that this number will be as high as 10 million by 2050 (Willyard, 2017; Amarasiri et al., 2020). Recently, it has been shown that antibiotic resistance genes (ARGs) carried by clinically drug-resistant pathogens originate from the environment (Forsberg et al., 2012; Yang et al., 2013; Fang et al., 2015; Huijbers et al., 2019). Therefore, ARGs have been widely considered as a new type of environmental pollutant and have become a research hotspot worldwide (Forsberg et al., 2012; Zhang et al., 2021).

Microorganisms in the soil can exchange signals by secreting low concentrations of antibiotics, which provide selective pressure for bacteria to obtain resistance (Adriaenssens et al., 2017). However, anthropogenic activities, such as sewage run-off, manure application, and reclaimed water irrigation, cause soil to become one of the largest ARG pools in the environment (Calero-Cáceres et al., 2019; Pu et al., 2020). The long-term use of antibiotics in the breeding industry provides a selective pressure for animal intestinal microflora. Therefore, the diversity and abundance of ARGs are increasing in the gut microbiome, resulting in livestock manure becoming an important source of ARGs in the environment (Wright and Poinar, 2012). Livestock manure is applied to farmland soil as organic fertilizers and significantly increases soil antibiotic resistance. A previous study found that the abundance of 63 ARGs subtypes in soil treated with manure was 192–28,000 times higher than that in the control soil (Chen et al., 2017). ARGs in soil can migrate into the endophytic and phyllospheric microbiomes of plants (Zhang et al., 2019; Pu et al., 2020; Guo et al., 2021), and subsequently enter the food chain, posing an eventual potential threat to human health (Forsberg et al., 2012; Ye et al., 2019).

Horizontal gene transfer (HGT) is the main mechanism underlying the enrichment and spread of ARGs in the environment (Debroas and Siguret, 2019; Lerminiaux and Cameron, 2019). ARGs can be horizontally transferred in three ways: conjugation mediated by mobile genetic elements, transformation by cell-free DNA, and phage-mediated transduction (Brown-Jaque et al., 2015; Mishra et al., 2021). Among the three HGT mechanisms, conjugation is generally considered the predominant mode of ARG dissemination in environmental settings (von Wintersdorff et al., 2016). Recently, a variety of ARG sequences were found in soil phage genomes, suggesting that phages may play an important role in mobile antibiotic resistance. However, the contribution of phages as reservoirs to HGT of ARGs has not been extensively explored. In this study, we reviewed the occurrence and distribution of ARGs in soilborne phage genomes (pARGs), the mode and characteristics of phage-mediated horizontal transfer of ARGs (transduction), and the contribution of phages to the mobile antibiotic resistome. In addition, the current research gap and future prospects are discussed. This article provides a scientific foundation for further expansion of the knowledge of the soil antibiotic resistome and controlling the spread of phage-mediated ARGs.

## Phages in soil

Phages, as bacterial viruses, are the most abundant and diverse entities in the biosphere, with a total population of approximately 10^31–33^, which is 10 times that of bacteria (Balcazar, 2014; Dion et al., 2020). The complete biological structure of a phage consists of a nucleic acid core (DNA or RNA) surrounded by an outer shell of a protein coat (capsid) with an estimated size of 20–200 nm (Jebri et al., 2021). Additionally, most phages belong to the order Caudovirales, which includes five families: Myoviridae, Siphoviridae, Podoviridae, Ackermannviridae, and Herelleviridae (Adams et al., 2017; Walker et al., 2019; Jebri et al., 2021). Phages are divided into two categories according to their life cycle: temperate and virulent phages. Temperate phages can be inserted into the host genome as a prophage and replicate with the host; this process is known as the lysogenic cycle (Ghosh et al., 2008). When external conditions change (such as UV radiation and heavy metal exposure), the prophage enters the lytic cycle using host nutrients to synthesize the offspring phage and kill the host (Ghosh et al., 2008; Zhong et al., 2021). Phages have a significant effect on the composition and diversity of microbial communities, biogeochemical cycles of global ecosystems, and bacterial evolution (Maganha De Almeida Kumlien et al., 2021; Lobo and Faciola, 2021).

Phages exist in various environments, among which the soil is an important habitat (Williamson et al., 2007; Ghosh et al., 2008). It is estimated that the number of phage particles in soil accounts for 10% of the total number of viruses globally (Vos et al., 2009; Cobian et al., 2016). High biodiversity and rich soil nutrition provide a suitable living environment for phages (Gomez and Buckling, 2011). Phages have been detected in various types of soil samples, including farmland, forests, wetlands, croplands, sand dunes, and even extreme environments, such as deserts and Antarctic soil (Williamson et al., 2007; Gomez and Buckling, 2011; Wang, 2019; Bezuidt et al., 2020; Chen et al., 2021). The abundance of phages in the different soil types are summarized in Table 1. Among the various soil samples that have been investigated by epi-fluorescence microscopy, the abundances of phages in farmlands and forests are the highest, reaching 10^9^/g, whereas the abundance of phages in deserts is nearly 3–6 orders of magnitude lower (Zablocki et al., 2016; Williamson et al., 2017; Trubl et al., 2018). With the continuous progress of metagenome sequencing and bioinformatics technology, the research of the “dark matter” of the microbial world—the Environmental virome (mainly composed of phages) has developed rapidly (Monaco and Kwon, 2017; Ofir and Sorek, 2018; Han et al., 2022). A viral metagenomic analysis of soil has suggested that viruses (phages) affect the microbial ecology (Ashelford et al., 2003; Dinsdale et al., 2008; Parsley et al., 2010; Cobian et al., 2016; Kavagutti et al., 2019). A recent study showed that phages play an important role in phosphorus metabolism, carbon metabolism, soil organic matter degradation, and polysaccharide binding after analyzing the permafrost thaw gradient (Emerson et al., 2018; Trubl et al., 2018; Emerson, 2019; Han et al., 2022). Additionally, soil properties and the bacterial community significantly affects the structure of the viral community (Chen et al., 2016, 2021). However, our understanding of soil phages is still relatively backward due to the high soil heterogeneity and the limitation of publicly available viral databases, especially regarding the contribution of phages to the soil antibiotic resistome (Larrañaga et al., 2018; Sun et al., 2018; Zhao et al., 2019).

**Table 1:** The viral abundance in different soil types.

Soil source	Virus (phage) abundance (gDW^−1^) ^a^	Method	References
Farmland	10^7^	Epifluorescence microscopy	Chen et al. (2014)
	10^8^–10^9^	Epifluorescence microscopy	Li et al. (2019) and Williamson et al. (2005)
	64,520 vOTU^b^	Illumina sequencing	Chen et al. (2021)
Desert	10^3^–10^7^	Epifluorescence direct counts	Gonzalez-Martin et al. (2013)
	10^8^	Epifluorescence direct counting	Williamson et al. (2007)
Forest	10^8^	Epifluorescence microscopy	Helsley et al. (2014)
	10^9^	Epifluorescence microscopy	Williamson et al. (2005)
Wetland	10^9^	Epifluorescence direct counting	Williamson et al. (2005, 2007)
Antarctica	10^8^	Epifluorescence direct counting	Williamson et al. (2007)
	11–33 vOTU	Ion Proton sequencing	Adriaenssens et al. (2017)

^a^gDW^−1^ (per gram dry weight of soil). ^b^vOTU (viral operational taxonomic units).

## The occurrence of soilborne pARGs

Research on ARGs in soilborne phages began relatively late compared with that in the aquatic environment. Ross and Topp (2015) quantified a set of ARGs from soilborne phages using quantitative polymerase chain reaction (qPCR) technology. In the following years, the occurrence and abundance of soilborne pARGs from different regions were investigated using qPCR technology (Anand et al., 2016; Larrañaga et al., 2018; Sun et al., 2018; Zhao et al., 2019). Recently, metagenomics has become a powerful tool to study the diversity and abundance of pARGs in soil (Enault et al., 2017). The diversity and abundance of soilborne pARGs are summarized in Table 2.

**Table 2:** Subtypes and abundance of antibiotic resistant genes (ARGs) in soilborne phage genomes.

ARGs type	ARGs subtype	Soil sample source	Location	ARGs abundance	Method	References
β-lactams	*bla*_TEM_	Animal farm	India	3.09%	PCR	Anand et al. (2016)
		Soil matrices	Spain	10^6^ –10^7^ GC/g	qPCR	Larrañaga et al. (2018)
	*bla*_*O*XA-2_	Animal farm	India	3.6%	PCR	Anand et al. (2016), Chen et al. (2017)
		Raw manure	Canada	10^1^–10 ^2^GC/ng DNA	Model (qPCR-based)	Ross and Topp (2015)
	*bla*_CTX-M_	Dairy farm	China	7.2 × 10^4^ –1.1 × 10^8^ copies/g	qPCR	Zhao et al. (2019)
		Soil matrices	Spain	10^3^ GC/g		Larrañaga et al. (2018)
Tetracycline	*tetA*	Animal farm	India	12.7%	PCR	Anand et al. (2016)
	*tetW*		India	9.1%		Anand et al. (2016)
	*tetC*	Greenhouse	China	10^2^ –10^3^ copies/g	qPCR	Sun et al. (2018)
	*tetE*			10^3^ –10^4^ copies/g		Sun et al. (2018)
	*tetG*			10^2^ –10^4^ copies/g		Sun et al. (2018)
	*tetM*			10^2^ –10^3^ copies/g		Sun et al. (2018)
	*tetO*			10^2^ –10^4^ copies/g		Sun et al. (2018)
	*tetX*			10^2^ –10^3^ copies/g		Sun et al. (2018)
	*tetT*	Organic fertilizer	China	/	Illumina sequencing	Chen et al. (2021)
	*tetQ*	Farmland soil	China	/	Illumina sequencing	Wang (2019)
	*tetN*					
	*tetV*					
	*tetL*					
	*tet36*					
	*ort(A)*					
Quinolones	*qnrA*	Soil matrices	Spain	10^1^–10^2^ GC/g	qPCR	Larrañaga et al. (2018)
	*oqxB*	Farmland	China	/	Illumina sequencing	Wang (2019)
Aminoglycosides	*aadA*	Raw manure	Canada	10^2^ GC/ng DNA	Model (qPCR-based)	Ross and Topp (2015)
	*armA*	Soil matrices	Spain	10^2^–10^4^ GC/g	qPCR	Larrañaga et al. (2018)
	*aac(3)-1*	Organic fertilizer	China	/	Illumina sequencing	Chen et al. (2021)
	*aad(9)*					
	*aac(2′)-1*	Farmland	China	/	Illumina sequencing	Wang (2019)
	*aadE*					
Sulfamethazine	*sul1*	Raw manure	Canada	10^5^ GC/ng DNA	Model (qPCR-based)	Ross and Topp (2015)
	*sul2*	Soil matrices	Spain	10^4^ GC/g	qPCR	Larrañaga et al. (2018)
Streptomycin	*strA*	Raw manure	Canada	10^1^ GC/ng DNA	Model (qPCR-based)	Ross and Topp (2015)
	*strB*			10^0^ GC/ng DNA		Ross and Topp (2015)
	*vgaD*	Farmland	China	/	Illumina sequencing	Wang (2019)
	*vatB*					
	*vatF*					
Chloramphenicol	*cmlA*	Dairy farm	China	3.5 × 10^5^–1.1 × 10^8^ copies/g	Real-time PCR	Zhao et al. (2019)
	*catB*	Organic fertilizer	China	/	Illumina sequencing	Chen et al. (2021)
	*CA*					
Trimethoprim	*dfrA1*					
	*dfrB2*					
	*dfrB6*					
	*dfrA12*					
	*afrA20*					
MLSB^a^	*vatB*					
	*macB*					
	*lmrC*	Farmland	China	/	Illumina sequencing	Wang (2019)
	*carA*							
	*lsaC*						
Vancomycin	*vanD*						
	*vanS*						
	*vanR*					
Rifamycin	*rpoB2*					
	*rphB*					
Pleuromutilin	*TaeA*					
Mupirocin	*mupB*					

*MLSB (Macrolide-Lincosamide-Streptogramin B)*.

### Detection methods of pARGs

The main methods for detecting pARGs are based on PCR technology, such as qPCR and droplet digital PCR (ddPCR), which can obtain absolute quantification information of specific ARG subtypes (Ross and Topp, 2015; Hu et al., 2022). Furthermore, recent metagenomic approaches provide a valuable tool for exploring the composition of the viral community, ARGs, and the correlations between them in different environmental settings (Chen et al., 2021). With the development of sequencing technology, more pARGs in soil will be unearthed, providing unprecedented opportunities to elucidate the mechanisms of mobile antibiotic resistance in soil (Chen et al., 2021). PCR technology and metagenomic sequencing have their own advantages. As a classical method for the absolute quantitative detection of ARGs in environmental samples and pure strains, PCR technology has the advantages of accuracy and rapidity (Hu et al., 2019a). In contrast, metagenomic sequencing does not depend on the microbial culture and screening process, and has the advantages of high specificity, sensitivity, and throughput (Hu et al., 2013; Hu et al., 2019a). Metagenomic sequencing facilitates the detection of more ARGs, which is conducive to the comprehensive understanding of the diversity of phage-mediated ARGs. The continuous innovation of sequencing technology can capture the phylogenetic and genetic diversity, fully understand the types, abundance, and transmission routes of phage-mediated ARGs, and predict the possible risk of antibiotic resistance and other potential threats to humans (Parsley et al., 2010; Bengtsson-Palme, 2017).

### The diversity of soilborne pARGs

ARG subtypes that confer resistance to 13 antibiotics (β-lactams, tetracycline, quinolone, aminoglycosides, sulfonamide, streptomycin, chloramphenicol, trimethoprim, MLSB, vancomycin, rifamycin, pleuromutilin, and mupirocin) were identified in phage genomes from various soil sources (Table 2). Among these, *tet* genes are the main types of soilborne pARGs. Tetracyclines are one of the most commonly used antibiotics in the livestock breeding industry, and their resistance genes are ubiquitous in agricultural soil. For example, Sun et al. (2018) detected six genes conferring resistance to tetracyclines in the phage fraction of greenhouse soil, including *tetC*, *tetE*, *tetG*, *tetM*, *tetO,* and *tetX.* In addition, large amounts of the *bla* gene have been identified in soilborne phages, including *bla*_TEM_, *bla*_CTX-M_, and *bla*_OXA-2_. Hu et al. (2022) analyzed a total of 25 ARG subtypes using ddPCR technology and demonstrated that the detection rate of soilborne pARGs with the application of organic fertilizer was 76%, whereas the rates of soilborne pARGs with the application of non-fertilizer and chemical fertilizer were only 68 and 72%, respectively. Chen et al. (2021) analyzed the viral DNA of soil samples using the Illumina NovaSeq 6000 platform and detected 16 unique ARG subtypes, including *catB*, *macB*, *vatB*, *dfrB2*, *dfrB6,* and so on. Another study detected 144 ARGs in soil viruses, which were divided into 12 categories according to the types of antibiotic resistance (Wang, 2019).

### The abundance of soilborne pARGs

Generally, the abundance of pARGs varies greatly between different soil types and ARG subtypes, ranging from 10^1^ to 10^8^ copies/g (Wang, 2019). A study of soilborne pARGs resistant to tetracycline found that the order of accumulative pARG abundance is as follows: efflux pump ARGs (*tetC* + *tetE* + *tetG*) > ribosome protection ARGs (*tetM* + *tetO*) > enzymatic modification ARG (*tetX*; Sun et al., 2018). As shown in Table 2, human activities, such as the application of organic fertilizer, affect the abundance of ARGs carried by soil phages. Another study also found that the relative abundance of pARGs in the soil receiving organic fertilizer treatment is significantly higher than that in soil receiving chemical fertilizer (*p* < 0.05; Wang, 2019). Hu et al. (2022) also found that soil receiving organic fertilizer has the highest total abundance of pARGs, reaching 1.6 × 10^3^ copies/g, and soil treated with non-fertilizer has the lowest pARG abundance (6.0 × 10^2^ copies/g).

## Characteristics of horizontal gene transfer in pARGs

The mechanisms of the horizontal transfer of ARGs are conjugation, natural transformation, and transduction (von Wintersdorff et al., 2016). Among them, plasmid-mediated conjugation has been identified as the main mechanism of HGT (Svara and Rankin, 2011). Some studies have found that ARGs resistant to amoxicillin and sulfanilamide are located in plasmids, with frequencies ranging from 10^−6^ to 10^−4^ (Musovic et al., 2010; Zhang et al., 2017). In addition, many plasmids have a wide-range of hosts, enabling ARGs to transfer among different biological species (Binh et al., 2008; Musovic et al., 2010). Transformation refers to the process in which the recipient bacteria directly obtain extracellular fragments of the donor bacteria to obtain new genetic traits (Hu et al., 2019a; Ding et al., 2021). However, the poor stability of extracellular DNA and low proportion of bacteria with natural transformation abilities make excludes transformation as the main mechanism of the horizontal transfer of ARGs (Nielsen et al., 2007). Recently, phage-mediated transduction has been considered as another mechanism for the horizontal transfer of ARGs (Subirats et al., 2016; Calero-Cáceres et al., 2019; Debroas and Siguret, 2019 Jebri et al., 2021).

**Figure 1:**
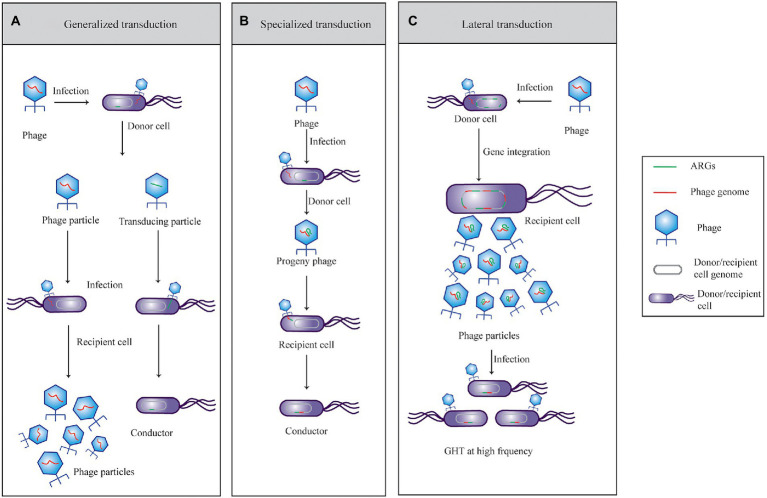
Horizontal transfer of phage-mediated antibiotic resistant genes through **(A)** general, **(B)** specialized, and **(C)** lateral transduction ARGs mediated by phages through transduction.

Phage-mediated transduction of ARGs has been documented for several bacterial species, including *Streptococcus pyogenes*, *Enterococci* sp., *Escherichia coli*, and *Salmonella* sp., and the transduced ARGs are mainly resistant to erythromycin, tetracycline, gentamicin, and β-lactam (Ubukata et al., 1975; Hyder and Streitfeld, 1978; Schmieger and Schicklmaier, 1999; Mazaheri et al., 2011; Billard-Pomares et al., 2014). Transduction can be divided into generalized or specialized transduction according to classical microbiology (Figures 1A,B). Generalized transduction that achieves a genetic trait is carried by two types of phage particles (temperate and virulent phages) from a donor cell to a recipient cell during lytic pathways (Sander and Schmieger, 2001). Phages can transfer any gene from one bacterium to another during this process (Penadés et al., 2014). Transduction particles wrap host DNA (including ARGs) by mispackaging, and their frequency is very low (10^−8^–10^−6^; Maganha De Almeida Kumlien et al., 2021). Another model of genetic material mediated by phages is specialized transduction, which can only be carried out by temperate phages during the late lysogenic cycle (Debroas and Siguret, 2019). More specifically, specialized transduction particles carry fixed genes of the bacterial chromosome located adjacent to the prophage (including ARGs) attachment site with a low occurrence frequency (10^−6^; Balcazar, 2014). The frequency of gene transfer due to “misloading” of host DNA fragments is very low, whether by general transduction or specialized transduction (Poté et al., 2003; Muniesa et al., 2013). The third recently discovered transduction model, lateral transduction, may be the most effective way for bacteria to obtain ARGs (Figure 1C; Chen et al., 2018; Fillol-Salom et al., 2021). Kenzaka et al. (2010) accurately measured the transduction frequency between *E. coli* mediated by phages using cycling primed *in situ* amplification-fluorescent *in situ* hybridization (CPRINS-FISH). The results revealed that the frequency of DNA transfer is 10^−4^–10^−3^, indicating that the phage-mediated exchange of genes occurs at an unexpectedly high frequency (Kenzaka et al., 2007, 2010). Phage-mediated HGT has many distinct characteristics, which can be summarized into the following three aspects.

### High occurrence frequency

A previous study showed that the transduction frequency of phages in aquatic environments is several orders of magnitude higher than expected (nearly 1%; Muniesa et al., 2013). Recently, Chen et al. (2018) found that lateral transduction in the process of HGT is mediated by the temperate phage of *Staphylococcus aureus*, proving that phages can transfer genes with extremely high frequency. The mechanism of this newly discovered transduction is DNA packaging initiated *in situ* from integrated prophages. Several hundred kilobases of the *S. aureus* genome, are packaged in phage heads at very high frequencies. *In situ* replication before DNA packaging creates multiple prophage genomes, such that lateral-transducing particles form during normal phage maturation, transforming parts of the *S. aureus* chromosome into hypermobile regions of gene transfer (Chen et al., 2018). With the deepening of our understanding of phages and their transduction mechanisms, the frequency of phage-mediated HGT in the environment may be much higher than expected. Overall, phages act as powerful agents to transfer bacterial chromosomal DNA to another organism *via* genetic transduction (Fillol-Salom et al., 2021).

### No direct contact between recipient and donor cells

Conjugation has been considered to play a major role in HGT and the consequent spread of ARGs and requires cell-to-cell contact (Muniesa et al., 2013). However, phage transduction does not need to meet the above conditions. Additionally, ARGs can be transferred by phages at different time points (Muniesa et al., 2013). Researchers have found that viral DNA can be transferred from hot springs (−82°C) to ice (−0°C), which strongly supports this view (Sano et al., 2004). Paez-Espino et al. (2016) identified that the virus can infect organisms from different phyla by linking the virus to the microbial host through the CRISPR spacer and transfer RNA matches. Furthermore, phage-mediated HGT is not restricted to microorganisms of the same species, but occurs between different species, genera, or even phyla (Muniesa et al., 2013).

### Persistence and broad time-scale

The genetic material of the phage is tightly surrounded by a protein coat called a capsid. Owing to their structure, phages thrive successfully in the environment and are resistant to natural and anthropogenic stressors such as enzymes, radiation, and antimicrobial substances (Muniesa et al., 2013; Göller et al., 2020). Durán et al. (2002) conducted an inactivation experiment of phages in rivers and showed that phages were more resistant than fecal coliforms and enterococci. As mentioned above, phage DNA will not be damaged in different extreme environments, and this conclusion is supported by the premise that the protein capsid is not broken (Sano et al., 2004). Overall, phages have stronger persistence and a broader time scale in the environment, making them more suitable as carriers for ARG transfer (Calero-Cáceres and Muniesa, 2016).

## The contribution of phages in the horizontal transfer of ARGs

Phages act as vectors for genetic exchange, facilitate reproduction (short-term) or promote microbial evolution (long-term). When phages containing ARGs move between different bacteria, it leads to the horizontal spread of ARGs and even the emergence of drug-resistant bacteria in soil (Kenzaka et al., 2007). Understanding the horizontal transfer mechanism of pARGs is important when investigating the mobile resistome, which is conducive to controlling the spread of ARGs.

### Research methods for phage-mediated transduction of ARGs

**Figure 2:**
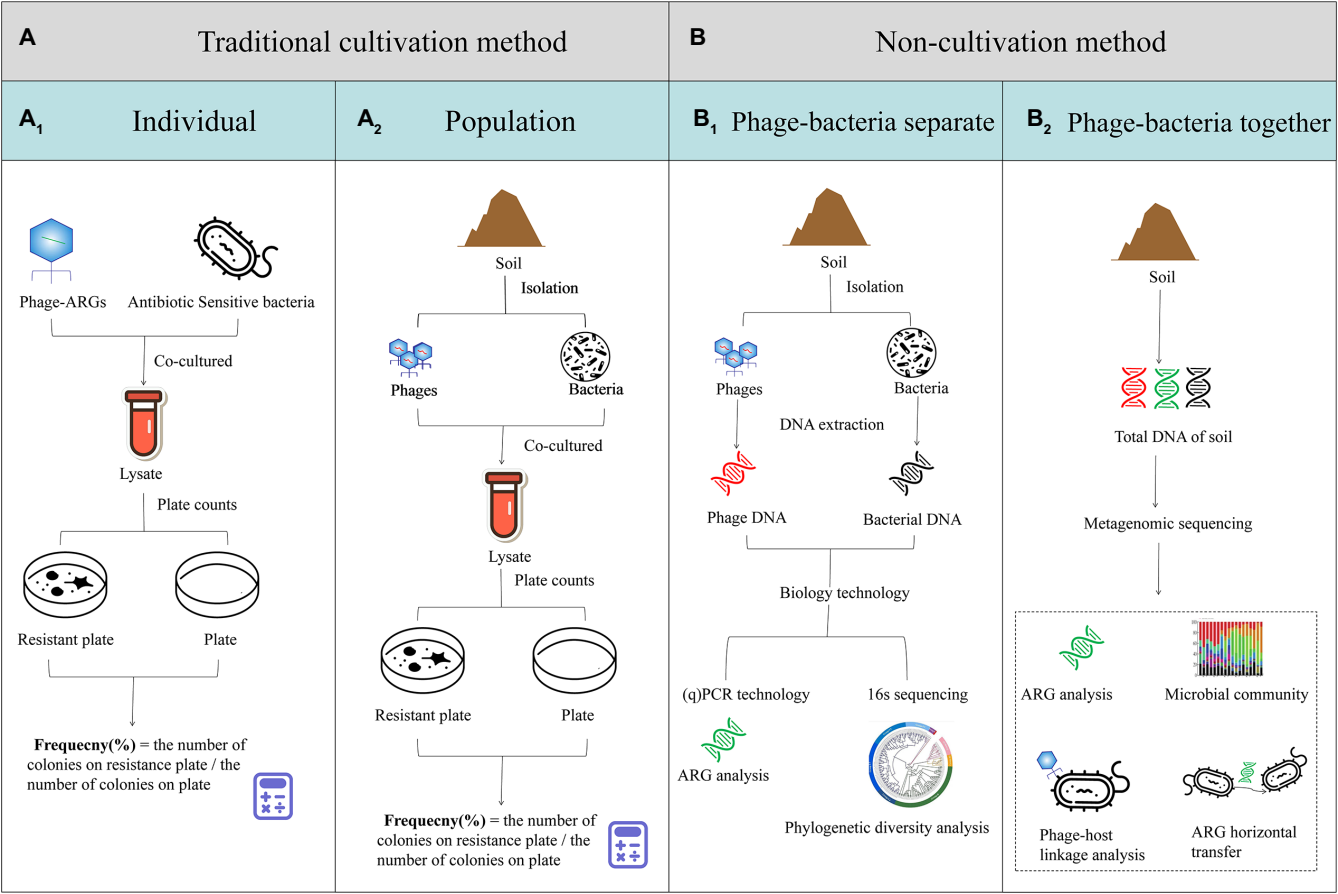
Research methods for phage-mediated transduction of antibiotic resistant genes (ARGs) *in vitro.* **(A)** Traditional cultivation methods using an individual phage containing ARGs co-cultured with antibiotic-sensitive bacteria **(A**_**1**_**)** or a soil sample from which the phage and bacteria populations were isolated and co-cultured **(A**_**2**_**)**. **(B)** Non-cultivation methods using soil samples from which the phage and bacteria populations are isolated using centrifugal filtration, their DNA is extracted and ARGs and phylogenetic diversity is analyzed through PCR or 16 s sequencing, respectively **(B**_**1**_**)**, or the total DNA of the soil is extracted analyzed through metagenomic sequencing **(B**_**2**_**)**.

A summary of the research methods for phage-mediated transduction of ARGs is shown in Figure 2. Before the advent of new generation sequencing technology, phage-mediated ARG transduction was mainly studied through cultivation-based methods (Bowring et al., 2022). Firstly, a specific phage containing ARGs is used to infect antibiotic sensitive bacteria (such as *E. coli*). After co-culturing, the lysate is diluted and plated on drug-free and drug-containing plates under appropriate conditions. The frequency of transduction is calculated by dividing the number of colonies on the drug-containing plate by that on the drug-free plate (Zeph et al., 1988; Mazaheri et al., 2011; Figure 2A_1_). Another research method uses the phage community as a whole to conduct *in vitro* transduction experiments. First, the bacteria and phages are isolated from environmental samples. After co-culturing, the plate count of the antibiotic-resistant bacteria (ARB) is carried out to investigate the rate of ARG transfer (Modi et al., 2013; Figure 2A_2_). With the emergence of molecular biotechnology, research on the horizontal transfer of ARGs is more profound and rigorous (Ross and Topp, 2015). Bacteria and phages are obtained from environmental samples by means of centrifugal filtration, and their DNA is extracted. Then, the abundance of ARG is analyzed through PCR and fluorescent labeling technology (Harrington et al., 2012; Larrañaga et al., 2018; Watson et al., 2018; Zhao et al., 2019; Figure 2B_1_). In recent years, genome sequencing technology has overcome the limitations of bacterial culture and microbial loss caused by isolation. As shown in Figure 2B_2,_ the transfer of ARGs is studied through the extraction of total environmental DNA, followed by the sequencing and analysis of the resistome and mobilome. The origin of AGRs and the role of phages in ARG transmission are explored through phylogenetic diversity and phage-host linkage analysis, respectively. In addition, the results of metagenomic or (virome) analysis can simultaneously reveal the microbial (viral) composition and function in soil, which contribute to the reliable risk assessment of ARG transmission through the source-tracking method and correlation analysis (Lekunberri et al., 2017; Chen et al., 2021).

### Horizontal transfer of ARGs mediated by phage

Many experimental studies have provided evidence that phages participate in the horizontal transfer of ARGs. Ross and Topp (2015) conducted transduction experiments using phages isolated from the soil to infect *E. coli* K-12. They found that a certain selective pressure (such as antibiotics) could promote the horizontal transfer of phage-mediated soilborne ARGs. Another study used phages-mediated ARGs to perform subculture experiments with *E. coli* as the host cells. By measuring the ARGs in the phage DNA before and after culture, it was proven that phages can reinfect the host and are important carriers for the transfer and transmission of ARGs in the environment (Larrañaga et al., 2018). Researchers have successfully transferred the *bla* gene in phage DNA into the *E. coli* genome using electroporation technology, which also suggests that phages are vehicles that transfer ARGs effectively (Colomer-Lluch et al., 2011).

However, our knowledge of phage-mediated HGT in soil lags far behind from what is known in other environmental media (e.g., water, animal gut) because of the high heterogeneity and rich biodiversity of the soil. A virome study showed that mouse intestinal phages were enriched with ARGs due to antibiotic treatment; these ARG-carrying phages infected intestinal bacteria, increasing the number of antibiotic-resistant bacteria (Modi et al., 2013). Shousha et al. (2015) found that phages isolated from chickens transduced the resistance of *E. coli* to various antibiotics (including ampicillin, tetracycline, and chloramphenicol). Another study demonstrated that phages released from *S.aureus* enable their hosts to acquire streptomycin-resistant genes from adjacent cells (Haaber et al., 2016).

### Factors affecting transduction of ARGs in soil

#### Antibiotics

Only a few studies were conducted to explore the relationship between antibiotics and the transfer of pARGs. A study on the gut virome showed that antibiotic treatment lead to the enrichment of pARGs. In addition, they assessed the proportion of resistant bacteria before and after phage infection with naive microbiota from mice gut as host by *in vitro* experiments, and found that the basal frequency (before infection) was lower than that microbiota infected with phages, especially in antibiotic-treated mice (Modi et al., 2013). These results demonstrated that antibiotic treatment enhanced the ability of phages to transmit resistance.

#### Physicochemical characteristics of soil

Numerous studies have demonstrated a positive correlation between the transfer of ARGs and the physicochemical characteristics of soil (Ji et al., 2012; Debroas and Siguret, 2019). Environmental factors, such as temperature, affected the dissemination of ARGs by exchange community boundaries (Iyer et al., 2013; Marti et al., 2014). Heavy metals could exert pressure on ARGs, thereby promoting the transfer of ARGs (He et al., 2020). Another study found that the concentration of Cu and Zn in soil was significantly positively correlated with the transduction of pARGs (Yang et al., 2021). Organic matter and heavy metals (Zn, Cr, and Cu) had a greater effect on pARGs than others factors, such as Hg, As, available phosphorus, available potassium, and total nitrogen (Hu et al., 2022). The concentrations of NH_4_-N and total phosphorus are positively correlated with two types of pARGs conferring resistant to tetracyclines and macrolide-lincosamide-streptogramin B (Yang et al., 2021). Random forest modeling and partial least squares path modeling results indicated that pH is a key factor affecting the occurrence of pARGs during the fermentation process (Xu et al., 2022).

#### Bacterial community

Soil bacteria and phages are crucial reservoirs of ARGs in the natural environment (Sun et al., 2018; Guo et al., 2021). The ARGs in phages are generally positively correlated with that of their bacterial hosts (Yang et al., 2021). Virus-host linkage analyses revealed that the phage-mediated ARGs are closely related to five bacterial phyla, including Firmicutes, Bacteroidetes, Proteobacteria, Crenarchaeota, and Planctomycetes (Chen et al., 2021). Yang et al. (2021) investigated the association between bacterial communities and pARGs and found that the bacterial community contributes to 16.7% of the variation in the pARG profiles. The results showed that *Terrisporobacter*, *Desulfovibrio*, and *Acinetobacter* are the main drivers impacting pARGs (Yang et al., 2021). Several recent studies have confirmed that the bacterial community is the main driver impacting the pARG profiles. Using the variance partitioning analysis (VPA), our previous study also found that the bacterial community contributes the most to pARG variation (10.81%; Hu et al., 2022).

#### Others

Recently, some studies have found that nano-metal oxide particles have an effect on the horizontal transfer of ARGs (Hu et al., 2019b; Otinov et al., 2020; Li and Zhang, 2022). Han et al. (2020) conducted an experiment using phage gM13 and *E.coli* exposed to nano-TiO_2_, and the results showed that nano-TiO_2_ increases the transduction frequency up to 4.5 fold compared to the control. TiO_2_ photoexcitation can drastically improve phage transduction efficiency 20.4-fold (Xiao et al., 2021). The transfer rate of ARGs mediated by nano-Al_2_O_3_ *via* a transduction-like pathway was 10^4^ times higher than that of the control. (Ding et al., 2021). The possible mechanisms of these nanomaterials promoting transduction are presumed to the increase of phage attachment on host cell surface, and cell membrane permeability. In the contrast, excessive UV irradiation led to a decrease in transduction efficiency (Xiao et al., 2021). Additionally, ionic liquid, as an environmental friendly compound, facilitates the horizontal transfer of ARGs (Wang et al., 2015a,b).

## Future perspectives

Phages play an important role in the antibiotic resistome of soil, and their contributions to the spread of ARGs should not be underestimated. However, there are still many uncertainties regarding the distribution characteristics of ARGs in the phage genome and the mechanism of phage-mediated HGT at different environmental sites. Therefore, additional studies are required to elucidate this and to subsequently support the inclusion of phages in monitoring, evaluation, and surveillance programs aimed at the emergence and spread of ARGs (Balcázar, 2020). The following gaps in research should be addressed:

1. The influence mechanism of the physicochemical properties and biological communities of soil on ARG transduction *via* phages is unclear. Although phages can mediate the spread of ARGs in agricultural soil and increase the number of antibiotic resistant bacteria under selective pressures, whether they can improve the antibiotic resistance of other indigenous bacteria and related mechanisms needs to be elucidated.
2. The research methodology on soilborne pARGs needs to be improved. Existing studies have only focused on ARGs in free phages, ignoring prophages in bacterial genomes, which leads to the inevitable underestimation of the contribution of phages to the environmental antibiotic resistome. In addition, the removal of bacterial contamination during phage DNA extraction is a challenge in the quantitative study of phage metagenomes in the soil (Göller et al., 2020). Although the development of sequencing technologies will undoubtedly help solve these problems, these approaches should be standardized to avoid misleading and incorrect conclusions.

## Conclusion

Phages are extremely abundant in soil and a large number of ARGs are present in their genomes. ARGs can be horizontally transferred through transduction at high frequencies, including specialized, generalized, and lateral transduction. Transduction can occur without contact between donor and recipient bacteria and is not limited by time and space. Moreover, there is increasing evidence that human activities, especially the application of organic fertilizers, lead to the enrichment and transmission of ARGs in soilborne phages. Therefore, the role of phages in the transfer of ARGs should not be ignored or underestimated. However, the contribution of phages to the transfer of ARGs has been relatively poorly studied because of the high heterogeneity and complexity of soils. With the advancement of sequencing technology, recent related research has been greatly developed. Additional studies are needed to elucidate the mechanisms and influencing factors contributing to the dissemination of ARGs *via* phages.

## Author contributions

YZ: writing — original draft preparation, visualization, and conceptualization. YG: writing — original draft preparation and conceptualization. TQ: writing — review and editing and funding acquisition. MG: writing — review and editing. XW: supervision, project administration, writing — review and editing, and funding acquisition. All authors have approved this work for publication.

## Funding

This work was supported by the National Natural Science Foundation of China (grant no. 32070089), Beijing Natural Science Foundation (grant no. 5222005), the Research Foundation of BAAFS (grant no. KJCX20200302), Beijing Postdoctoral Science Foundation (grant no. 202222104), and Postdoctoral Science Foundation of BAAFS (grant no. 2021ZZ006).

## Conflict of interest

The authors declare that the research was conducted in the absence of any commercial or financial relationships that could be construed as a potential conflict of interest.

## Publisher’s note

All claims expressed in this article are solely those of the authors and do not necessarily represent those of their affiliated organizations, or those of the publisher, the editors and the reviewers. Any product that may be evaluated in this article, or claim that may be made by its manufacturer, is not guaranteed or endorsed by the publisher.

## Abbreviations

HGT: Horizontal gene transfer
ARGs: Antibiotic resistance genes
AMGs: Auxiliary metabolic genes
qPCR: Quantitative polymerase chain reaction
SOM: Organic matter in soil
pARGs: Antibiotic resistance genes carried by phages
